# A protocol-based treatment for ruptured abdominal aortic aneurysm contributed to improving aorta-related mortality: a retrospective cohort study

**DOI:** 10.1186/s12872-023-03473-8

**Published:** 2023-09-01

**Authors:** Yusuke Takei, Masahiro Tezuka, Shunsuke Saito, Takeshi Ogasawara, Masahiro Seki, Takashi Kato, Yasuyuki Kanno, Shotaro Hirota, Ikuko Shibasaki, Hirotsugu Fukuda

**Affiliations:** 1 Department of Cardiac and Vascular Surgery, Dokkyo Medical University Graduate School of Medicine, 880 Kitakobayashi, Mibu-Machi, Simotuga-gun, Tochigi, 321-0293 Japan; 2 Mathematics and Statistics Section, Department of Fundamental Education, Dokkyo Medical University, 880 Kitakobayashi, Mibu-Machi, Simotuga-gun, Tochigi, 321-0293 Japan

**Keywords:** Ruptured abdominal aortic aneurysm, Open surgical repair, Endovascular aortic aneurysm repair, Endovascular occlusion balloon, Abdominal compartment syndrome

## Abstract

**Background:**

Recent guidelines state that improving the survival rate of patients with ruptured abdominal aortic aneurysm (rAAA) requires a protocol or algorithm for the emergency management of these patients. We aimed to investigate whether introducing a protocol treatment for rAAA improves clinical outcomes compared with the pre-protocol strategy.

**Methods:**

At our institution, 92 patients treated for rAAA between June 2008 and August 2022 were retrospectively analyzed. In 2014, the protocol-based treatment was introduced comprising a transfer algorithm to shorten the time to proximal control, use of an endovascular occlusion balloon, strict indications for endovascular aortic aneurysm repair (EVAR) or open surgical repair, and perioperative care, including for abdominal compartment syndrome (ACS). Clinical outcomes were compared between the protocol and pre-protocol group, including operative status, all-cause mortality, and rAAA-related death at 30-day, in-hospital, and 1-year postoperative follow-ups.

**Results:**

Overall, 52 and 40 patients received the protocol-based and pre-protocol treatments, respectively. EVAR was more frequently performed in the protocol group. The rate of achieving time to proximal control was significantly faster, and the transfusion volume was lower in the protocol group. ACS occurred more frequently in the protocol group with a higher EVAR. No difference was found in all-cause mortality between the two groups. The protocol group exhibited fewer rAAA-related deaths than the pre-protocol group during the following time points: 30 days (9.6% vs. 22.5%), during the hospital stay (11.5% vs. 30.0%), and 1 year (14.5% vs. 31.5%).

**Conclusions:**

The protocol-based treatment improved the survival rate of patients with rAAA.

**Supplementary Information:**

The online version contains supplementary material available at 10.1186/s12872-023-03473-8.

## Background

A ruptured abdominal aortic aneurysm (rAAA) is one of the most severe, life-threatening conditions. According to estimates, the total incidence of rAAA is 5.6 per 100,000 inhabitants [1]. Recently, with the advent of endovascular aortic aneurysm repair (EVAR), treatment options for abdominal aortic aneurysms have expanded, and EVAR has been performed in patients with rAAA. A recent study on EVAR for rAAA showed a mortality rate of 21% [2]. Large randomized controlled trials comparing open surgical repair (OSR) and EVAR for rAAA, such as IMPROBE [3], AJAX [4], ECAR [5], and a Japanese nationwide study [6], have shown comparable outcomes or advantages for EVAR regarding activities of daily living and cost-effectiveness [7]. Therefore, recent guidelines considered EVAR as the first line of treatment for rAAA [8, 9]. However, it is impossible to perform EVAR in all patients because only 47% of all rAAA cases meet the mandatory anatomical conditions to perform EVAR [10]. As the recent guidelines state, improving the survival rate requires a protocol or algorithm for the emergency management of patients with rAAA [8, 11]. The protocol consisted of the following: the roles of each staff member were clearly defined, the patient was transferred from the emergency room (ER) to the operating room (OR) at an early stage, hemodynamics were stabilized using an endovascular occlusion balloon (EOB), and either OSR or EVAR was performed quickly and smoothly. Nevertheless, protocol-based treatments are gradually becoming more widespread, and only a few publications have discussed their results. In accordance with this trend, our institution has implemented treatment protocols to improve survival rates. This study aimed to compare the outcomes before and after introducing this protocol for rAAA to demonstrate its usefulness.

## Methods

### Study design

This observational, single-center, retrospective cohort study was approved by the Institutional Review Board of Dokkyo Medical University (protocol number R-63-7 J) on October 24, 2022. The study was performed in accordance with the ethical standards laid down in the 1964 Declaration of Helsinki and its later amendments. The opt-out declaration form was in the public domain. The requirement for informed consent was waived by the Institutional Review Board of Dokkyo Medical University because of the retrospective nature of the study. A patient was excluded from the study when withdrawal was requested.

### Patients

Consecutive patients treated for rAAA, including iliac aneurysm rupture at Dokkyo Medical University Hospital from June 2008 to August 2022, were included in this study. The following cases were excluded from this study: impending rupture, infectious aortic aneurysm rupture, rupture of anastomotic pseudoaneurysm, rupture due to endoleak after EVAR, or rupture of residual iliac artery aneurysm in patients who underwent OSR or EVAR; patients with cardiac arrest on arrival without a return of spontaneous circulation following cardiopulmonary resuscitation for ˃ 40 min; or no rise in blood pressure (> 60 mmHg) or return of spontaneous circulation after EOB insertion. Since 2014, the protocol outlined below has been implemented, and an EVAR-first strategy has been adopted. On the other hand, before the protocol introduction, OSR was generally performed, and EVAR was only performed after waiting for the stent graft to arrive if the patient’s hemodynamics were stable and when their aortic anatomy was suitable for EVAR. Regarding EOB prior to protocol implementation, only patients with shock underwent EOB (RESCUE BALLOON®, Tokai Medical Products Inc., Aichi, Japan), which was implanted through the brachial artery and blocked in the descending aorta.

### Protocol-based treatment for rAAA in dokkyo medical university hospital

The protocol-based treatment for rAAA was introduced in 2006 by Metha et al. [12], and good outcomes were reported in 2013 by Ogino et al. [13]. Currently, this protocol is recommended per treatment guidelines [8, 10, 11] and has become a standard approach. After consulting with acute care physicians, anesthesiologists, operating room staff, and surgeons, our institution implemented these protocols. Modifications were made to adapt them to the specific conditions at our hospital.

The first version of the protocol was implemented at our institution in 2014 based on a previously reported protocol [12, 13] (Additional File 1). In 2016, the hybrid operation room (HOR) was fully equipped next to the ER, and the second version was implemented. This led to a shorter transfer time and more accurate EVAR with excellent image quality (Artis Q Ceiling; Siemens Healthineers, Erlangen, Germany). The current version (version 3.0) was updated in 2019 to modify the patients’ transfer algorithm and the EVAR technique using n-butyl-2-cyanoacrylate (NBCA) to prevent bleeding from the rupture site due to typeII endoleak. Perioperative care for abdominal compartment syndrome (ACS) has been increasingly recognized with the increase in the number of EVAR procedures. ACS is among the worst and most frequently lethal complications for rAAA following EVAR [14] that leads to multiple-organ failure (MOF), increased airway pressure, and decreased cardiac output. ACS management was tightened, the criteria for decompression laparotomy were established, and a peritoneal negative-pressure therapy system was applied.

### Patients’ transfer algorithm and the EOB method

**Fig. 1:**
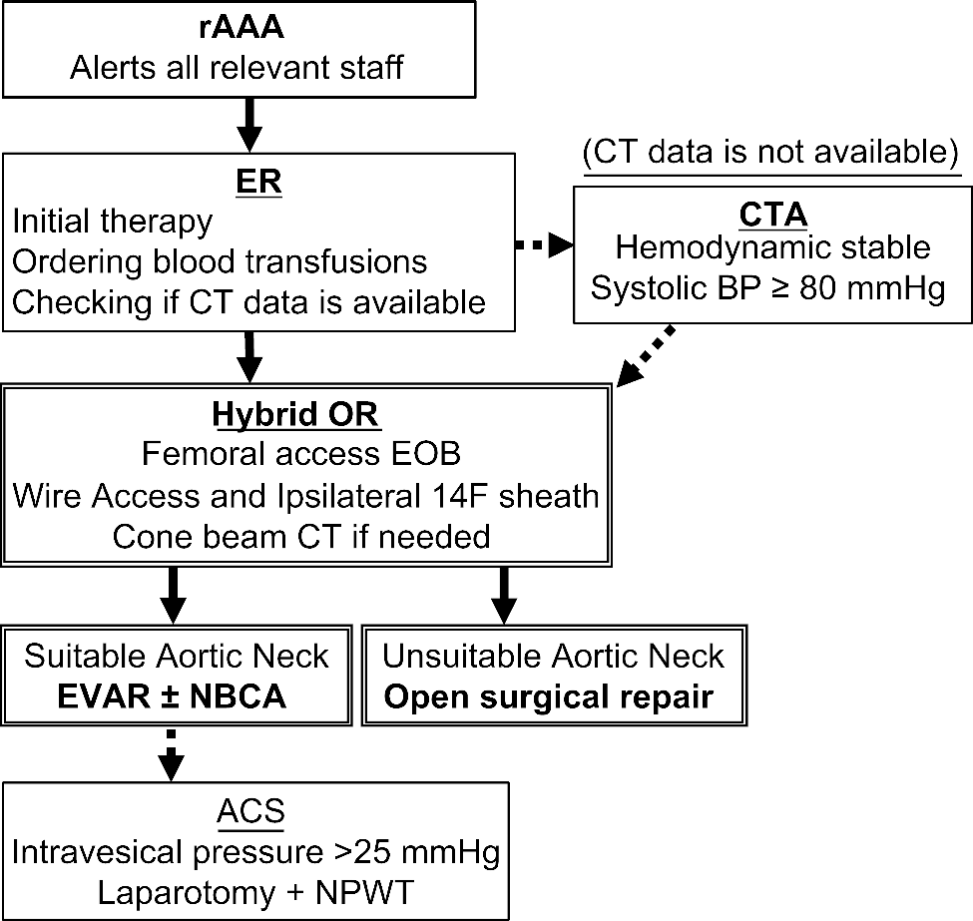
The latest version of the endovascular-first protocol algorithm for rAAA. rAAA, ruptured abdominal aortic aneurysm; CTA, computed tomographic angiography; OR, operation room; EOB, endovascular occlusion balloon; EVAR, endovascular aneurysm repair; NBCA: n butyl-2-cyanoacrylate; ACS, abdominal compartment syndrome; NPWT, negative-pressure wound therapy; CT, computed tomography

The latest version showed that a patient is directly transferred from the ER to the HOR as soon as the diagnosis of rAAA is confirmed by abdominal echography or CT data taken at the previous hospital. If there are no CT data and the patient’s circulation is stable, CT angiography can be performed (Fig. 1). Once the patient was transferred to the HOR, local anesthesia was administered to the groin, an 8 F short sheath was inserted into the common femoral artery under echo guidance, and a closure device (Perclose ProGlide™, Abbott, Chicago, IL, USA) was used, or punctured by cut down fashion. A 5 F catheter (Kumpe Access Catheter, Cook Medical, Bloomington, USA or TEMPO®, Cordis, Hialeah, USA) was inserted into the descending aorta using a 0.035-inch floppy wire (RADIFOCUS™, TERUMO, Tokyo, Japan), which was subsequently replaced with a stiff wire (EGoist, MEDICO’S HIRATA, Tokyo, Japan) and a 14 F GORE® Drysheal Flex Introducer sheath (W. L. Gore & Associates, Newark, USA). Subsequently, an EOB (Reliant Stent Graft balloon catheter, Medtronic, Minneapolis, MN, USA) was inflated in the descending aorta depending on blood pressure. A cone beam CT was then performed to determine the necessity of the surgical procedure. In many cases, EVAR was performed under general anesthesia. However, the procedure could be performed under local anesthesia if the patient’s anatomy allowed for it, the patient’s vital signs were stable, and the patient was able to follow our instructions. In contrast, general anesthesia was administered if the OSR was determined, and the EOB was inflated intermittently until aortic clamping was completed.

### EVAR indications and procedure

The stent grafts used were usually Gore Excluder C3 (W. L. Gore & Associates, Newark, USA) or Endurant IIs (Medtronic, Minneapolis, MN, USA). Excluder C3, trunk-ipsilateral leg endoprostheses of all aortic diameter sizes with a length of 120 mm, Endurant IIs, bifurcated stent grafts of all proximal diameter sizes with a length of 124 mm, and all sizes of Excluder cuffs and legs were all accessible in the hospital. EVAR was performed if the proximal landing length was appropriate for instructing the use of each device unless the infrarenal aorta was highly curved. We used Excluder as our first choice because of the risk of type IV endoleak with Endurant IIs. During EVAR, another balloon can be inserted from the contralateral side in addition to the previously inserted EOB to minimize bleeding from the rupture site; the EOB is inflated immediately after the main body deployment. The latest version of the protocol calls for sealing the NBCA-lipiodol mixture into the rupture site from the catheter left in the aneurysm sac if angiograms identify a rupture site after the completion of the stent graft.

### Definition of ACS and indication for decompression laparotomy

ACS was diagnosed when the trans-bladder intra-abdominal pressure (IAP) was > 20 mmHg after EVAR or increased when measuring IAP every 2 h in the ICU [15]. Decompression laparotomy was indicated for patients with IAP ≥ 25 mmHg [16] who did not show improvement despite treatments such as optimizing fluid balance, deep sedation, and using neuromuscular blockade. If decompression laparotomy was performed, the abdominal surface was covered with negative pressure wound treatment (NPWT) with ABTHELLA™ (3 M, Minnesota, US). After the blood had clotted and dried for several days, the abdomen was closed. At the same time, the retroperitoneal hematoma was removed, and the lumbar artery was sutured to reduce the aneurysm. If intestinal necrosis was observed, an urgent resection was added.

### Endpoints and clinical outcomes

The primary endpoint was rAAA-related death defined as follows: intraoperative death; MOF during the perioperative course related to the procedures; re-rupture; ischemic colitis with ACS and sepsis related to graft infection. The secondary endpoint was all-cause mortality. Moreover, other operative data such as the patient’s transport time (time from the ER to the OR or HOR, namely, time to OR), the time to aortic clamp with an aortic clamper or EOB (time from the ER to aortic clamp, namely, time to proximal control), the time to completion of the graft replacement or EVAR, transfusion dose, and perioperative complications were compared between the two groups. Red blood cell (RBC) transfusions were performed when hemoglobin was < 8.0 g/dL, and fresh frozen plasma (FFP) was administered when fibrinogen was < 150 mg/dL or in the case of massive transfusions.

### Data collection

Data were obtained from patient charts stored in the hospital database. In addition, the authors gathered information over the phone from patients who were discharged, moved to other hospitals, and those who could no longer attend outpatient clinics, or their relatives. Variables with many missing data were excluded, and only those with few missing data (within 5%) were included.

### Statistical analysis

Continuous variables were presented as mean (standard deviation [SD]) or median (interquartile range [IQR]), and categorical variables were described as numbers (%). After testing for normality, the comparison of means and medians between the groups was analyzed using the unpaired Student’s t-test and the Mann–Whitney U-test for normally and non-normally distributed parameters, respectively. Categorical variables were compared using chi-square or Fisher’s exact tests, and time-to-event studies were compared using the log-rank test with Kaplan–Meier product limit estimates. The Cox proportional-hazards model was used to evaluate the primary endpoint. We analyzed risk using the existence of protocol intervention and occurrence of ACS as covariates after adjusting for confounding variables such as sex, a Glasgow Scale score > 85 for preoperative status, including age, and a Fitzgerald score of ≥ 3 for the severity of rAAA. All analyses were performed using the SPSS version 27 software (IBM Corp., Armonk, NY, USA), and a two-tailed *p* < .05 was considered statistically significant.

## Results

### Patient demographics

**Fig. 2:**
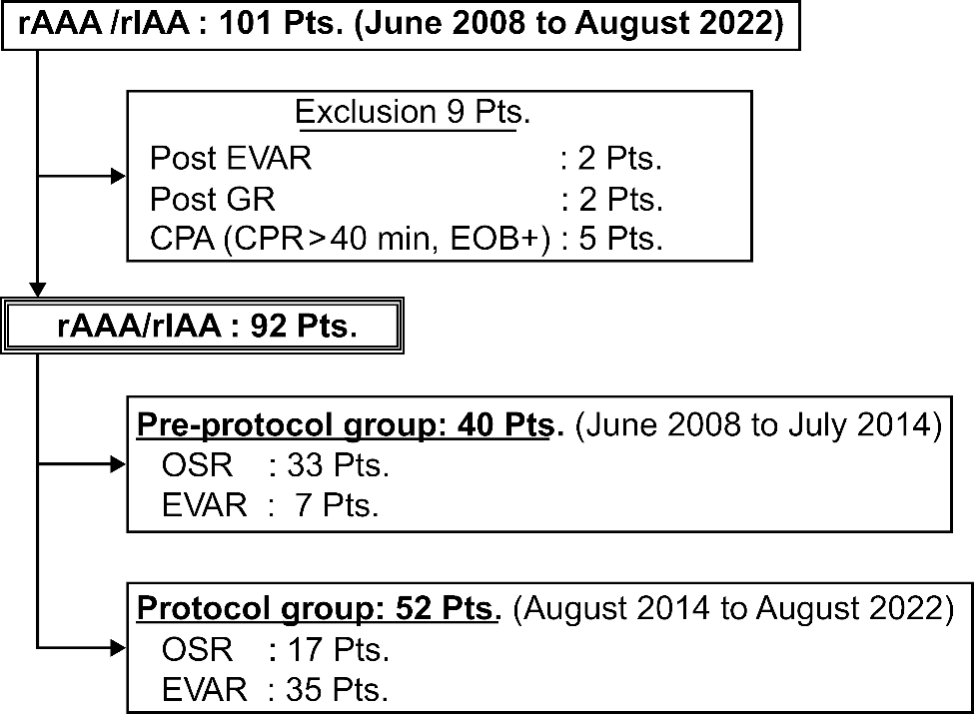
Inclusion and exclusion criteria of the study cohort. rAAA, ruptured abdominal aortic aneurysm; rIAA, ruptured iliac artery aneurysm; EVAR, endovascular aneurysm repair; GR, graft replacement; CPA, cardiac pulmonary arrest; CPR, cardiopulmonary resuscitation; OSR, open surgical repair; EOB, endovascular occlusion balloon

We consecutively treated 101 patients with rAAA with or without iliac artery aneurysms at our institution between June 2008 and August 2022. According to our exclusion criteria, nine patients were withdrawn, including two with re-rupture due to endoleak after previous EVAR, two with ruptured pseudoaneurysm after OSR for abdominal aortic aneurysm, and five without return of spontaneous circulation following cardiopulmonary resuscitation. Finally, we enrolled 92 patients in the study. Of these, 40 patients were treated before the protocol, and 52 underwent each procedure based on the protocol after 2014. In the pre-protocol group, 7 (17.5%) and 33 (82.5%) patients were treated with EVAR and OSR, respectively, while 35 patients (67.3%) in the protocol group were suitable for EVAR, and the remaining 17 underwent OSR (Fig. 2). Table 1 summarizes the preoperative characteristics of the two groups. The average age was 75 ± 9.3 years, and males accounted for 79.3% of the total, with no remarkable differences between the groups. For comorbidities, patients with chronic kidney disease were significantly more prevalent in the protocol group than in the pre-protocol group (32.5% vs. 5%, *p* < .01); however, no other comorbidities differed remarkably. The mean diameter of rAAA was 69.3 ± 13 mm, and 34.8% and 7.6% of the patients had common iliac and internal iliac aneurysms, respectively, with no difference between the two groups. In addition, Fitzgerald ≥III accounted for 66.3% of all patients and 81.5% of the patients with a Glasgow scale > 85, which indicates a critical preoperative condition; however, no difference was found between the two groups.

**Table 1:** Comparison of the patient preoperative demographics between the pre-protocol and protocol groups

	Total (N = 92)	Pre-Protocol (N = 40)	Protocol (N = 52)	*p*-value
Age, years	75.0 [9.3]	75.8 [8.5]	74.5 [9.9]	0.51
Male, n (%)	73 (79.3)	29 (72.5)	44 (84.6)	0.15
IHD, n (%)	15 (16.3)	6 (15.0)	9 (17.3)	0.76
CVD, n (%)	8 (8.7)	4 (10.0)	4 (7.7)	0.48
COPD, n (%)	6 (6.5)	1 (2.5)	5 (9.6)	0.17
CKD, n (%)	19 (20.7)	2 (5.0)	17 (32.7)	< 0.01
History of laparotomy, n (%)	16 (17.4)	7 (17.5)	9 (17.3)	0.98
Diameter of AAA, mm	69.3 [13.0]	70.5 [12.2]	68.4 [13.7]	0.44
CIAA, n (%)	32 (34.8) One side: 21 Both sides: 11	13 (32.5) One side: 8 Both sides: 5	14 (26.9) One side: 8 Both sides: 6	0.85
IIAA, n (%)	7 (7.6) One side:7 Both sides:0	1 (2.5)	6 (11.5)	0.10
Fitzgerald ≥ III, n (%)	61 (66.3)	28 (70.0)	33 (63.5)	0.51
Glasgow scale > 85, n (%)	75 (81.5)	33 (82.5)	42 (45.7)	0.83

Data are presented as the mean [standard deviation], or n (%)

IHD, ischemic heart disease; CVD, cerebral vascular disease; COPD: Chronic obstructive pulmonary disease; CKD, chronic kidney disease; AAA, abdominal aortic aneurysm; CIAA, common iliac artery aneurysm; IIAA, internal iliac artery aneurysm

### Patient transfer time and time to proximal control

Table 2 shows the status of patient transfer from the ER to the OR before and after the protocol. Approximately 72% were transferred from other clinics to our institution; however, no difference was found between the two groups. Time to OR was considerably shorter in the protocol group than that in the other group (median 30.5 min vs. 67.5 min, *p* < .01). Time to proximal control was also much shorter in the protocol group than that in the pre-protocol group (median 66.5 min vs. 118.0 min, *p* < .01). In addition, all patients were inserted with an EOB in the protocol group, and the number of EOB inflations was higher than in the other group.

**Table 2:** Comparison of patient transfer status and EOB between the pre-protocol and protocol groups

	Total (N = 92)	Pre-Protocol (N = 40)	Protocol (N = 52)	*p*-value
Transfer from other hospitals, n (%)	66 (71.7)	29 (72.5)	37 (71.2)	0.88
Time to OR, min	41.0 [23–74.7]	67.5 [39.3–99.3]	30.5 [18.0–47.3]	< 0.01
Time to proximal control, min	88.5 [54.3–126.3]	118.0 [90.5–185.5]	66.5 [43.0–99.0]	< 0.01
EOB inflation, n (%)	40 (43.5)	9 (22.5)	40 (43.5)	< 0.01

Data are presented as the median [interquartile range] or n (%)

OR, operating room; EOB, endovascular occlusion balloon

### Operative outcome

Table 3 presents the intraoperative blood transfusion volumes and complications. More patients in the protocol group did not require a large amount of transfusion of RBC (1,540 mL vs. 2,520 mL, *p* < .01) or FFP (960 mL vs. 1,800 mL, *p* < .01). However, ACS occurred more frequently in the protocol group. Other complications were similar between the two groups. Regarding procedure time, no difference was found in the completion time of graft replacement, but EVAR was shorter in the protocol group (Additional File 2). Regarding the characteristics of patients with ACS in the protocol group, a tendency for a larger preoperative aneurysm diameter and greater blood transfusion volume was found in patients with ACS than in those without ACS (Additional File 3).

**Table 3:** Comparison of the amount of transfusion and operative complications between the pre-protocol and protocol groups

	Total (N = 92)	Pre-Protocol (N = 40)	Protocol (N = 52)	*p*-value
RBC, mL	1960 [875–3360]	2520 [1400–4130]	1540 [560–2450]	< 0.01
FFP, mL	1200 [630–2820]	1800 [1200–3540]	960 [0–2400]	< 0.01
DIC, n (%)	9 (9.8)	6 (15.0)	3 (5.9)	0.17
ACS, n (%)	14 (15.2)	2 (5.0)	12 (23.1)	0.02
Ischemic colitis, n (%)	12 (13.0)	3 (7.5)	9 (17.3)	0.19
MNMS, n (%)	3 (3.3)	2 (5.0)	1 (1.9)	0.57
Newley HD, n (%)	12 (13.0)	8 (20.0)	4 (7.7)	0.07

Data are presented as the median [interquartile range] or n (%) RBC, red blood cells; FFP, fresh frozen plasma; DIC: disseminated intravascular coagulation; ACS; abdominal compartment syndrome; MNMS; myonephropathic metabolic syndrome; HD: hemodialysis

### Endpoints

**Fig. 3:**
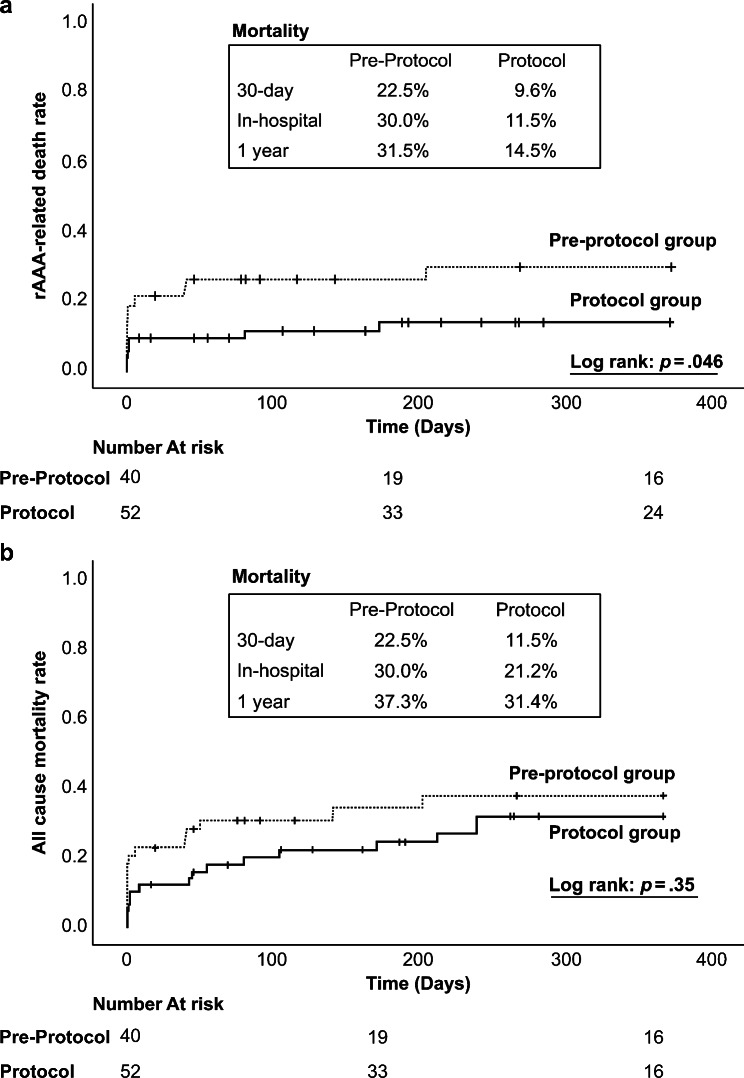
Kaplan–Meier curves illustrating rAAA-related death (a) and all-cause mortality (b) between the pre-protocol and the protocol group. rAAA, ruptured abdominal aortic aneurysm

The median follow-up time after the procedure for the pre-protocol and protocol groups was 127 days and 272 days, respectively. A significant difference in rAAA-related death was observed between the two groups (*p* = .046) at 30-day, in-hospital, and 1-year post operation (9.6%, 11.5%, and 14.5%, respectively, in the protocol group) (Fig. 3a). However, it did not reach statistical significance in all-cause mortality between the two groups (*p* = .35) (Fig. 3b). Thirty-day, in-hospital, and 1-year postoperative mortality in the protocol group were 11.5%, 21.2%, and 31.4%, respectively. In the pre-protocol group, most patients experienced disseminated intravascular coagulation due to massive intraoperative bleeding or in the early postoperative period, resulting in MOF (Additional File 4). In addition, analysis of rAAA-related death using the procedure showed no difference in EVAR but a lower mortality rate in the protocol group for OSR (Additional File 5a, b). Cox regression analysis adjusted for confounding factors identified the following factors associated with rAAA-related death: protocol-based treatment (hazard ratio = 0.19, *p* < .01) and ACS (hazard ratio = 11.45, *p* < .00) (Table 4).

**Table 4:** Cox regression analysis of rAAA-related death

	Hazard ratio	95% CI	*p*-value
Protocol-based treatment	0.19	0.06–0.57	0.003
ACS	11.45	3.67–35.59	< 0.00

This model was adjusted for preoperative confounding variables, such as sex, Fitzgerald classification ≥ III, and Glasgow scale score > 85. rAAA: ruptured abdominal aortic aneurysm; ACS: abdominal compartment syndrome; CI, confidence interval.

## Discussion

The main findings of the present study are as follows: (1) clearly defining the roles of the staff involved in the treatment and optimizing the patient transfer algorithm facilitated rapid transportation to the OR/HOR. The use of EOB as a hemodynamic stabilization method ensured stable hemodynamics throughout the procedure, regardless of OSR or EVAR. This method contributed substantially to improved OSR mortality after implementing the protocol. (2) While selecting EVAR based on appropriate aortic morphologic assessment reduced intraoperative blood transfusions and promoted early postoperative recovery, patients requiring massive blood transfusions or experiencing prolonged operative times remained more susceptible to ACS. This finding underscores the negative impact of ACS on the prognosis of patients with rAAA. (3) However, overall, implementation of the treatment protocol led to a significant improvement in aortic-related deaths.

The protocol-based treatment for rAAA was proposed by Metha et al. [12]. Numerous studies have investigated the surgical outcomes of EVAR and OSR in rAAA [3–6]. Although EVAR has a slight advantage [17, 18], its efficacy remains controversial. As a result, each institution has adopted a treatment policy of either EVAR or OSR first, and favorable results have been documented [19, 20]. Nevertheless, the fundamental guiding principle of these treatment policies is the protocol-based treatment mentioned above. Until now, the introduction of HORs and the establishment of medical networks have resulted in notable improvements at each institution [21]. The well-organized protocol-based therapy has dramatically improved treatment outcomes since recent studies have reported a 30-day mortality of 14.3–23.2% [12, 13, 22, 23]. In this study, we updated the protocol using the HOR and cone beam CT to reduce the patient transport time to take a CT scan, although the guidelines clearly state that a CT scan should be performed if the patient’s condition is stable [8, 9].

As suggested in the SVS practice guideline [11], achieving a door-to-intervention time of < 90 min for patient transport is just as critical as for ischemic heart diseases. This guideline defines the door as the initial medical contact and the intervention as arterial access for EOB. Although this criterion did not directly improve mortality in a previous study [24], early intervention is believed to be preferable. This guideline’s preoperative management and patient transfer considerations consist of three phases: diagnosis and initial care, rapid transfer from an initial institution to a vascular center, and vascular team intervention, each lasting 30 min. Our study aimed to improve the last phase. Our latest version of the transfer algorithm would shorten the patient transfer from the ER to the OR or HOR and the completion of the proximal control in the protocol group rather than the pre-protocol. However, as shown in Table 2, even in the protocol group, it took a long time from when the patient arrived at the OR or HOR until the EOB was inserted. We did not achieve this phase’s guideline recommendation of 30 min or less. Although there is a technical factor to accessing the femoral artery, there is a waste of time before the start of the surgery, and we need to improve this problem.

Mortality within the first 48 postoperative hours after aortic aneurysm rupture repair, including the operation, still dominates [25]. The cause of this mortality is associated with hemorrhagic shock [26]. Endovascular procedures make aortic occlusion to stabilize a patient’s vitals with a balloon catheter, which is more feasible than the traditional method of clamping the descending aorta through a left thoracotomy or direct clamping of the upper abdominal aorta. Before the protocol implementation, 8/14 (57.1%) of the patients, who had been controlled by direct aorta clamping, died during or immediately after the surgery due to massive bleeding and subsequent coagulation dysfunction, or MOF. Previous reports have shown that EOB can prevent this severe condition [27, 28]. Depending on the type of device, it is controversial whether to insert the device in an antegrade or retrograde fashion from the viewpoint of influencing subsequent procedures. We retrogradely inserted the device because it is easier to maneuver from the operative field and using a balloon catheter with a Gore Dry sheath enables precise aortic blockade. Another reason is that antegrade fashion carries a risk of peripheral embolization from the aortic arch or descending aorta [27]. Using an EOB would prevent hypotension during general anesthesia and intraoperative bleeding during OSR because no rAAA-related death was observed in the group of patients following the OSR protocol.

The introduction of this protocol resulted in an improved mortality rate. However, protocol-based therapy leads to more EVAR, which poses specific complications. Here, two deaths were caused by re-rupture after EVAR (type III and Ib endoleaks). Coagulation dysfunction after laparotomy caused two deaths due to long-lasting type II or IV endoleaks. For the former, we ensured that an expert endovascular surgeon was in charge of pre-sizing, and for the latter, we included a method of sealing the rupture site with NBCA. Regarding ACS in the protocol group, the incidence of ACS was 23.1%, which is higher than the reported incidence of ACS of 11.5% [29]. One patient died early because of intestinal necrosis caused by a delay in diagnosis. Overall, 5 of 12 patients developed ischemic colitis with necrosis, and all died of complications following colectomy. Furthermore, three of these five cases were performed concomitantly with internal iliac artery coil embolization, which took a long time to complete. We retrospectively reviewed cases of ACS and found that patients with more giant aneurysms were selected for EVAR; they received more blood transfusions than those who did not develop ACS. Heavy blood transfusion is believed to increase the risk of ACS [16, 29], and the postoperative persistence of a sizeable intraperitoneal mass causes the development of ACS. One reason for the numerous transfusions was the maintenance of blood pressure. To prevent this, it will be necessary to share with our team that a policy of permissive hypotension is recommended [9, 11]. The other reason was that it took a long time to complete the EVAR due to our insistence on challenging EVAR. This problem will be improved with the technical improvement of EVAR. Although ACS treatment has shown improvements [30], it has not yet addressed ischemic colitis and necrosis, which are directly related to mortality at an early stage. Therefore, future studies are required to identify the risk factors for the onset of ACS and subsequent ischemic colitis or necrosis and to develop countermeasures against these risk factors. As reported previously [31, 32], flexible sigmoidoscopy should be performed aggressively in at-risk patients to identify ischemic colitis early.

This study has some limitations. First, it was a retrospective study conducted at a single center with a small cohort, constrained by the current protocol-based treatment introduced in 2014, making sample size alteration before protocol implementation infeasible. Although increasing the post-protocol sample size was necessary for better detection power, obtaining a similar size as the pre-protocol was time-consuming because of the low number of rAAA treatments per year, making further augmentation impractical. Despite observing a significant difference in rAAA-related deaths between the groups in this small sample, there was no significant difference in all-cause mortality. Limited statistical power may have contributed to this discrepancy. Second, this study had selection and information biases. Selection bias occurred due to a lack of in-hospital stock of stent grafts before protocol introduction, leading to OSR being the inevitable treatment method and causing an overrepresentation of OSR cases in the pre-protocol group. Information biases included increased awareness among healthcare providers about patient transportation after implementing the protocol, early detection of ACS after EVAR due to strict criteria, and progressive upgrades of the protocol over the study period. These modifications could potentially bias the results, leading to an overestimation of the effectiveness of the protocol-based treatment. Third, many patients were transferred from other hospitals, making it crucial to consider both the rupture onset time and transfer duration, as they may affect mortality. Unfortunately, obtaining all relevant data on these events was not possible, highlighting the limitations of this study. Additionally, the coronavirus outbreak after 2020 might have impacted the transportation algorithm, leading to delays in reaching the HOR owing to waiting for test results. Patients needing surgery in complete isolation rooms to prevent infection could also have influenced surgical outcomes. These factors should be considered when interpreting the study results.

## Conclusions

Despite stepwise modifications, the implementation of the protocol allowed for prompt transfer from the ER to the OR, stabilization of blood pressure using EOB, and immediate surgery. Since anatomical indications were sufficient, EVAR was selected, whereas EOB was used with OSR to ensure procedural safety. This team protocol-based approach to rAAA improved the survival rate of patients with rAAA.

### Electronic supplementary material

Below is the link to the electronic supplementary material.

Additional File 1. First edition of the protocol algorithm for rAAA rAAA, ruptured iliac artery aneurysm; ER, emergency room; CTA, computed tomographic angiography; OR, operation room, EOB; endovascular occlusion balloon; EVAR, endovascular aneurysm repair Additional File 2. Differences between groups by surgical technique. Additional File 3. Comparison of patient demographics in the development of ACS in the protocol group. Additional File 4. Cause of death. Additional File 5. Kaplan–Meier curves for each technique in each group, where (a) is for OSR and (b) is for EVAR. OSR, open surgical repair; EVAR, endovascular aneurysm repair.

## Data Availability

The datasets used and/or analyzed during the current study are available from the corresponding author on reasonable request.

## Abbreviations

ACS: Abdominal compartment syndrome
EVAR: Endovascular aortic aneurysm repair
rAAA: Ruptured abdominal aortic aneurysm
OSR: Open surgical repair
ER: Emergency room
OR: Operating room
EOB: Endovascular occlusion balloon
NBCA: N-butyl-2-cyanoacrylate
MOF: Multiple-organ failure
HOR: Hybrid operation room
CT: Computed tomography
IAP: Intra-abdominal pressure
NPWT: Negative pressure wound treatment
RBC: Red blood cell
FFP: Fresh frozen plasma
SD: Standard deviation
IQR: Interquartile range
