# Supplementary material for: A protocol-based treatment for ruptured abdominal aortic aneurysm contributed to improving aorta-related mortality: a retrospective cohort study
Source: BMC Cardiovasc Disord. 2023 Sep 1;23:436. doi: 10.1186/s12872-023-03473-8 (PMC10474727; doi:10.1186/s12872-023-03473-8)
Supplement: Supplementary file 1 — Additional File 1. First edition of the protocol algorithm for rAAA rAAA, ruptured iliac artery aneurysm; ER, emergency room; CTA, computed tomographic angiography; OR, operation room, EOB; endovascular occlusion balloon; EVAR, endovascular aneurysm repair Additional File 2. Differences between groups by surgical technique. Additional File 3. Comparison of patient demographics in the development of ACS in the protocol group. Additional File 4. Cause of death. Additional File 5. Kaplan–Meier curves for each technique in each group, where (a) is for OSR and (b) is for EVAR. OSR, open surgical repair; EVAR, endovascular aneurysm repair. [file 12872_2023_3473_MOESM1_ESM.docx]

**Supplementary Materials**


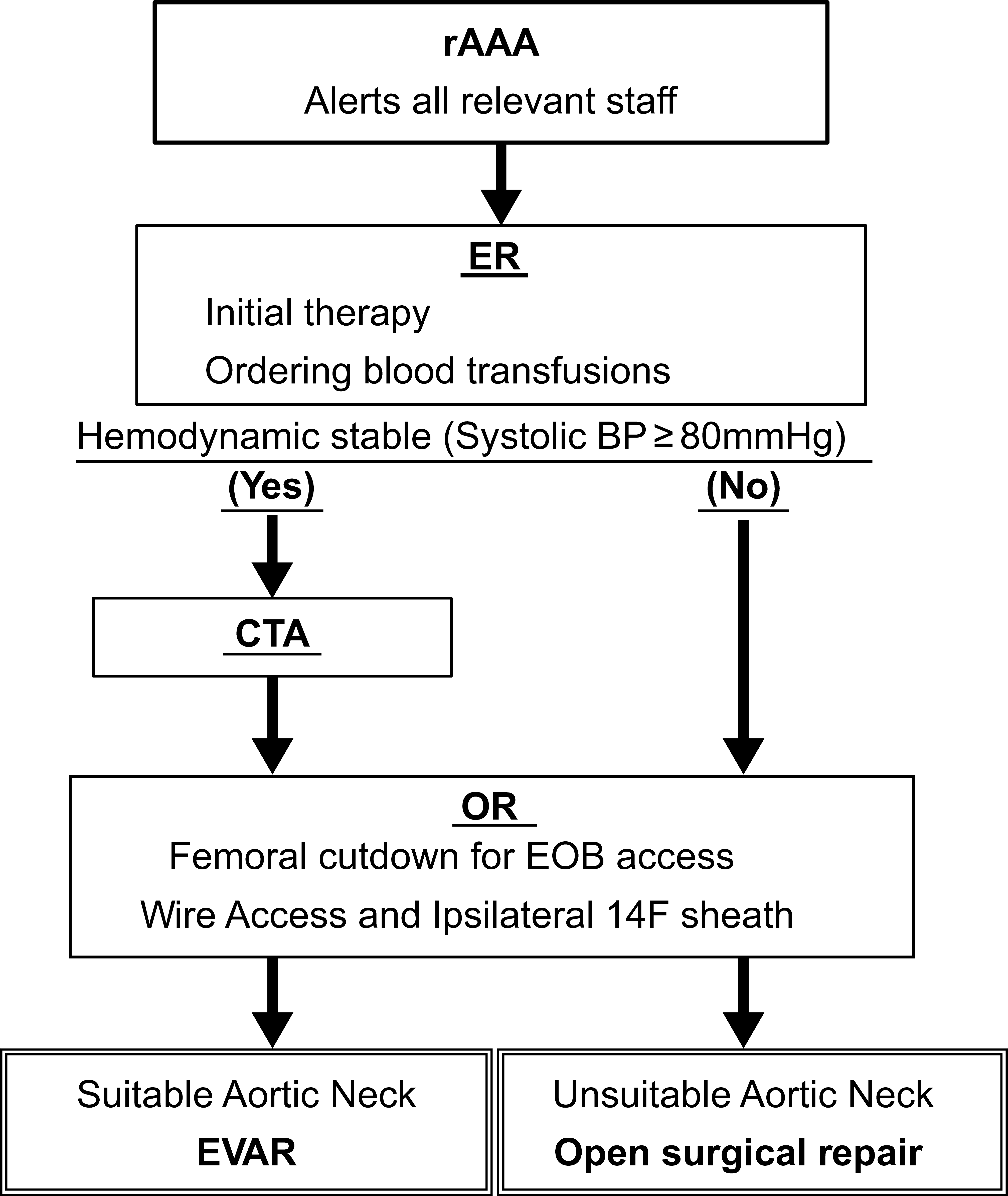


**Additional File 1**. First edition of the protocol algorithm for rAAA

rAAA, ruptured iliac artery aneurysm; ER, emergency room; CTA, computed tomographic angiography; OR, operation room, EOB; endovascular occlusion balloon; EVAR, endovascular aneurysm repair

**Additional File 2**. Differences between groups by surgical technique

|  | OSR (N= 50) | |  | EVAR (N=42) | |  |
| --- | --- | --- | --- | --- | --- | --- |
|  | Pre-Protocol (N=33) | Protocol (N=17) | p-value | Pre-Protocol (N=7) | Protocol (N=35) | p-value |
| EOB inflation, n (%) | 7 (21.2) | 13 (76.5) | <.00 | 2 (28.6) | 18 (51.4) | .41 |
| OSR completion time, min | 245.0 [96.6] | 246 [71.0] | .96 |  |  |  |
| EVAR completion time, min |  |  |  | 254.3 [66.9] | 162 [71.1] | .03 |
| Local anesthesia, n (%) |  |  |  | 0 | 7 (20.0) | .32 |
| NBCA injection, n (%) |  |  |  | 0 | 15 (42.9) | .03 |
| Open conversion, n (%) |  |  |  | 2 (28.6) | 4 (11.4) | .25 |

Data are presented as mean [standard deviation] or n (%). OSR, open surgical repair; EVAR: endovascular aneurysm repair; EOB: endovascular occlusion balloon, NBCA: n-butyl-2-cyanoacrylate

**Additional File 3**. Comparison of patient demographics in the development of ACS in the protocol group

|  | Non-ACS (N=40) | ACS (N=12) | P-value |
| --- | --- | --- | --- |
| Diameter of AAA, mm | 66.0 [13.3] | 76.6 [11.8] | .02 |
| Fitzgerald ≥ Ⅲ, n (%) | 24 (60.0) | 9 (75.0) | .50 |
| Glasgow scale > 85, n (%) | 33 (82.5) | 9 (75.0) | .68 |
| Time to proximal control, min | 80 [43.5, 105.5] | 51 [34.8, 75.3] | .11 |
| EVAR, n (%) | 23 (57.5) | 12 (100) | <.01 |
| EVAR completion time, min | 135.0 [107.0, 176.0] | 157.0 [123.8, 235.0] | .30 |
| RBC, ml | 1120 [560, 1960] | 3080 [2030, 3640] | <.01 |
| FFP, ml | 720 [0, 2040] | 2160 [1800, 3120] | <.01 |
| Ischemic colitis, n (%) | 4 (10.0) | 5 (41.7) | .02 |
| MNMS, n (%) | 0 | 1 (8.3) | .23 |
| Newley HD, n (%) | 2 (5.0) | 2 (16.7) | .22 |
| In-hospital mortality, n (%) | 4 (10.0) | 7 (58.3) | <.01 |

Data are presented as the mean [standard deviation], median [interquartile range], or n (%).

RBC, red blood cells; FFP, fresh frozen plasma; DIC: disseminated intravascular coagulation; ACS; abdominal compartment syndrome; MNMS; myonephropathic metabolic syndrome; HD: hemodialysis; EVAR: endovascular aneurysm repair

**Additional File 4**. Cause of death

| Causes of death in the pre-protocol group | | | |
| --- | --- | --- | --- |
| Case | Cause of death | Days from the operation to the date of death | rAAA-related death |
| 1 | Hemorrhagic shock | 0 | Yes |
| 2 | Acute subdural hematoma | 140 | No |
| 3 | Anastomotic pseudoaneurysm | 201 | Yes |
| 4 | Multiple organ failure | 39 | Yes |
| 5 | Multiple organ failure | 40 | Yes |
| 6 | Myocardial infarction | 50 | No |
| 7 | Myonephropathic metabolic syndrome, Multiple organ failure | 5 | Yes |
| 8 | Hemorrhagic shock | 0 | Yes |
| 9 | Hemorrhagic shock | 0 | Yes |
| 10 | Hemorrhagic shock | 0 | Yes |
| 11 | Hemorrhagic shock | 0 | Yes |
| 12 | Multiple organ failure | 1 | Yes |
| 13 | Hemorrhagic shock | 0 | Yes |
| 14 | Hemorrhagic shock | 0 | Yes |
| Causes of death in the protocol group | | | |
| Case | Cause of death | Days from the operation to the date of death | Cardiovascular related death |
| 1 | Gastrointestinal bleeding | 42 | No |
| 2 | Hemorrhagic shock | 0 | Yes |
| 3 | Acute subdural hematoma | 8 | No |
| 4 | Myonephropathic metabolic syndrome, Multiple organ failure | 2 | Yes |
| 5 | Pneumonia | 104 | No |
| 6 | Thoracic aortic aneurysm rupture | 238 | No |
| 7 | Died of old age | 238 | No |
| 8 | Graft infection | 170 | Yes |
| 9 | Suffocation | 54 | No |
| 10 | Multiple organ failure | 0 | Yes |
| 11 | Multiple organ failure | 79 | Yes |
| 12 | Lung cancer | 211 | No |
| 13 | Hemorrhagic shock | 1 | Yes |
| 14 | Sepsis without graft infection | 44 | No |
| 15 | Multiple organ failure | 2 | Yes |





**Additional File 5**. Kaplan–Meier curves for each technique in each group, where (a) is for OSR and (b) is for EVAR

OSR, open surgical repair; EVAR, endovascular aneurysm repair; rAAA, ruptured abdominal aortic aneurysm
